# Genetic and Epigenetic Mechanisms of Longevity in Forest Trees

**DOI:** 10.3390/ijms241210403

**Published:** 2023-06-20

**Authors:** Anastasia Y. Batalova, Konstantin V. Krutovsky

**Affiliations:** 1 Genome Research and Education Center, Laboratory of Forest Genomics, Department of Genomics and Bioinformatics, Institute of Fundamental Biology and Biotechnology, Siberian Federal University, 660036 Krasnoyarsk, Russia; batalova0910@mail.ru; 2 Department of Forest Genetics and Forest Tree Breeding, Faculty of Forest Sciences and Forest Ecology, Georg-August University of Göttingen, Büsgenweg 2, 37077 Göttingen, Germany; 3 Center for Integrated Breeding Research (CiBreed), Georg-August University of Göttingen, Albrecht-Thaer-Weg 3, 37075 Göttingen, Germany; 4 Laboratory of Population Genetics, N.I. Vavilov Institute of General Genetics, Russian Academy of Sciences, Gubkin Str. 3, 119333 Moscow, Russia; 5 Scientific and Methodological Center, G.F. Morozov Voronezh State University of Forestry and Technologies, Timiryazeva Str. 8, 394036 Voronezh, Russia

**Keywords:** *Dracaena*, *Ficus*, forest trees, genetic mechanisms, *Ginkgo*, longevity, *Pinus*, *Populus*, *Pseudotsuga*, *Larix*, *Quercus*, *Welwitschia*

## Abstract

Trees are unique in terms of development, sustainability and longevity. Some species have a record lifespan in the living world, reaching several millennia. The aim of this review is to summarize the available data on the genetic and epigenetic mechanisms of longevity in forest trees. In this review, we have focused on the genetic aspects of longevity of a few well-studied forest tree species, such as *Quercus robur*, *Ginkgo biloba*, *Ficus benghalensis* and *F. religiosa*, *Populus*, *Welwitschia* and *Dracaena*, as well as on interspecific genetic traits associated with plant longevity. A key trait associated with plant longevity is the enhanced immune defense, with the increase in gene families such as *RLK*, *RLP* and *NLR* in *Quercus robur,* the expansion of the *CC-NBS-LRR* disease resistance families in *Ficus* species and the steady expression of R-genes in *Ginkgo biloba*. A high copy number ratio of the *PARP1* family genes involved in DNA repair and defense response was found in *Pseudotsuga menziesii*, *Pinus sylvestris* and *Malus domestica*. An increase in the number of copies of the epigenetic regulators *BRU1/TSK/MGO3* (maintenance of meristems and genome integrity) and *SDE3* (antiviral protection) was also found in long-lived trees. CHG methylation gradually declines in the *DAL 1* gene in *Pinus tabuliformis*, a conservative age biomarker in conifers, as the age increases. It was shown in *Larix kaempferi* that grafting, cutting and pruning change the expression of age-related genes and rejuvenate plants. Thus, the main genetic and epigenetic mechanisms of longevity in forest trees were considered, among which there are both general and individual processes.

## 1. Introduction

The genetic control of growth and longevity are fundamental problems in plant biology. Trees, with their dominance of apical growth throughout their entire life and the vast differences in lifespan, are promising objects for the investigation of these problems. Many tree species live for several hundred years, and some of them have the world’s longest lifespan, reaching several millennia. For example, the longest-lived non-clonal living gymnosperm tree known to date, a bristlecone pine (*Pinus longaeva*) tree growing in the White Mountains in eastern Sierra Nevada (Inyo County, CA, USA), is almost 5000 years old [1].

Several factors have been proposed to explain the longevity of trees, such as sustained growth over a long period of time, resistance to biotic and abiotic stress (major mortality factors, especially in larger trees), the accumulation and transmission of adaptive somatic mutations and an increase in the number and copy number of genes, especially those associated with repair and immune protection. Genetically controlled traits that are common among long-lived tree species are vegetative vigor (sprouting, epicormic and reiteration branches), long reproductive period and resistance to stress, such as long droughts, and pests, including insects, bacteria, viruses and fungi [2].

One of the “secrets” of the longevity of long-lived trees is the constant renewal of living structures and the preservation of an intact vascular system, despite the reduction in the proportion of living structures in relation to the total biomass. This is achieved by building new tissue on top of the dead tissue through the constant development of the secondary phloem [3].

This review discusses the genetic aspects of longevity for such woody plants as *Quercus robur*, *Ginkgo biloba*, *Ficus benghalensis* and *F. religiosa*, as well as interspecific genetic traits associated with the plant lifespan.

## 2. *Quercus*

In the recently sequenced genome of *Quercus robur*, a comparison of nearly 600 gene families with 15 other dicot species showed that the *RLK*, *RLP* and *NLR* gene families involved in immune defense are among the most numerous and diverse of all gene families [4]. It has been suggested that a large number of defense-related genes may be a distinctive genomic trait of long-lived trees, conferring resistance to a variety of pathogens encountered over their centuries-long life.

How does a tree that lives for decades or centuries effectively interact with microbiomes and resist pathogenic microorganisms? The mechanism of dynamic specific pathogen recognition in long-lived plants can be explained by expanding our current understanding of plant defense genes. It was assumed that in addition to the physiological responses of trees, a tripartite genomic approach is used for protection, including (1) larger numbers of diverse resistance and defense response genes (R-genes), (2) genomic architecture characterized by the tight clustering of defense response genes, and R-genes in particular, and (3) the accumulation of adaptive somatic mutations over a long lifespan [5]. A study of the oak genome [4] confirmed all three of these proposed genomic traits that may contribute to successful tree longevity.

**Figure 1:**
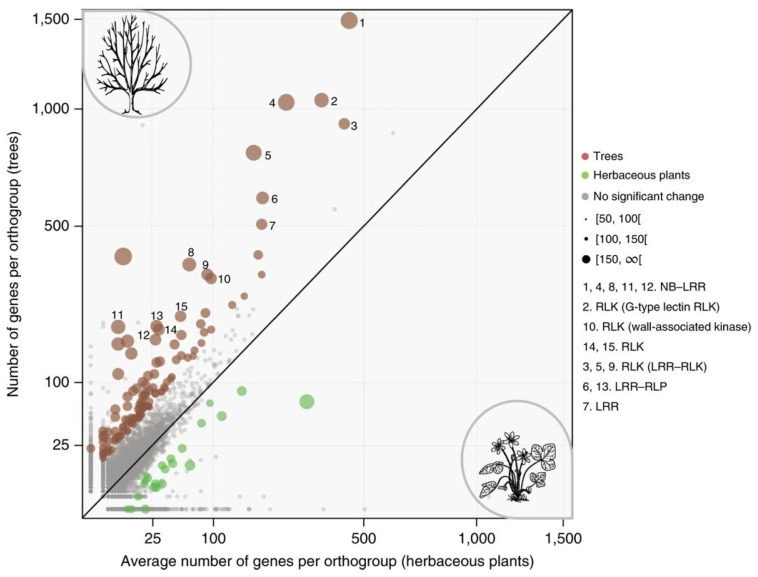
Scatter plot showing orthogroups expanded in trees and herbaceous plants. Numbers in square brackets associated with circle sizes stand for -log(*p*-adjust), where *p*-adjust is the *p*-value of the binomial test adjusted for multiple testing (Figure 4b in [4]).

A similar enrichment of gene families associated with receptor-mediated signaling is also observed in other trees. For instance, an extension of the family of R-related genes has also been found in other woody perennials, such as *Carica papaya*, *Citrus climentina*, *Eucalyptus grandis*, *Malus domestica*, *Populus trichocarpa*, *Prunus persica*, *Theobroma cacao* and *Vitis vinifera,* in comparison with herbaceous plant species (Figure 1) [4].

The adaptive value of R-genes was studied in extended oak orthogroups, and 260 sites for positive selection were identified, with more than 78% of them located in LRR domains. It has been found that positive selection mainly targeted four amino acids of the hypervariable region of the characteristic LXXLXLXX β-sheet/β-turn structure of LRRs, which has been implicated in protein–protein interactions [4].

Interestingly, the high expression of plant defense genes with leucine-rich nucleotide binding site repeats (NBS-LRRs) can often be costly and even lethal to plant cells [6]. Therefore, plants implement several mechanisms to control the level of NBS-LRR protective gene transcripts, including various microRNAs targeting NBS-LRRs as negative regulators of transcription [6].

## 3. *Ginkgo biloba*

**Figure 2:**
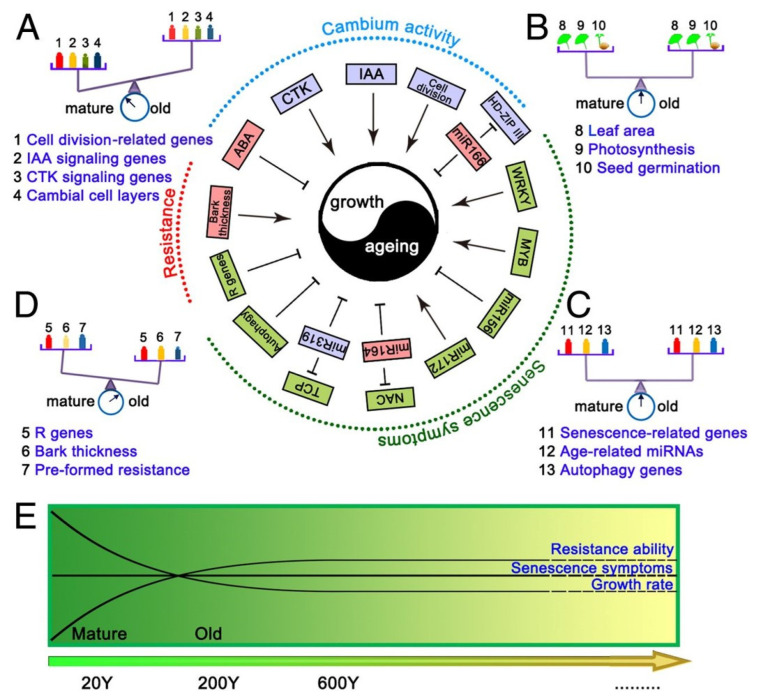
Schematic representation of the balance between aging and growth. The blue, red and green boxes represent decreased, increased and invariant index values in old trees, respectively. (**A**) Balance diagram; the balance between growth and aging is maintained by decreased cambium activity. (**B**,**C**) Old *G. biloba* trees lack senescence symptoms. (**D**) Resistance mechanisms delay senescence in old trees. The color gradation shows the values of indices. (**E**) Variation in growth rate, senescence symptoms and the resistance ability with age (modified Figure 7 in [3]).

*Ginkgo biloba* is a relict gymnosperm species and the only living species in the Ginkgophyta division. Some of its specimens can live for more than three or even four thousand years. Wang et al. [3] studied trees between 15 and 667 years old and showed that older trees show similar leaf areas, leaf photosynthesis efficiency and seed germination rates. Transcriptomic analysis has shown that the extensive expression of genes associated with preformed and inducible defense mechanisms most likely contributes to the remarkable longevity of this species (Figure 2) [3].

The vascular cambium of the oldest trees, although forming less xylem, does not show signs of aging. The following age-related changes in the vascular cambium of *G. biloba* are observed from 15 to 667 years:

- average basal area increment (BAI) continuously increased with aging, showing that the lateral meristem can retain indeterminacy in old trees;
- the indole-3-acetic acid (IAA) concentration in cambial cells decreased with age, whereas the content of abscisic acid (ABA) increased significantly;
- cell division-, cell expansion- and differentiation-related genes exhibited significantly lower expression in old trees;
- disease resistance-associated genes retained high expression in old trees, along with genes associated with synthesis of preformed protective secondary metabolites [3].

Notably, a comprehensive evaluation of the expression of genes related to autophagy, senescence and age-related miRNAs, together with an analysis of leaf photosynthetic efficiencies and seed germination rates, demonstrated that the old trees are still in a healthy, mature state, and senescence is not manifested at the whole-plant level [3].

Taken together, these results indicate that long-lived trees have evolved compensatory mechanisms to maintain a balance between growth and aging. This includes continued cambial divisions, high expression of resistance-associated genes and the continued synthetic capacity of pre-formed protective secondary metabolites (Figure 2) [3].

## 4. *Ficus*

In the Indian subcontinent, two species of ficus, *Ficus benghalensis* and *F. religiosa*, are the best-known examples of the longest-lived trees. *F. benghalensis* is an evergreen, semi-epiphyte tree with a lifespan of several centuries (named “Thimmamma Marrimanu”, native to South India, 550 years old [7,8]). *F. religiosa*, also known as the “sacred fig”, is a large semi-epiphyte, deciduous tree with an average lifespan of 900–1500 years (a tree native to the Buddhist Temple at Anuradhapura, Sri Lanka, 2217 years old, http://www.rmtrr.org/oldlist.htm, accessed on 14 February 2023). The genomes of both species are diploid (2n = 26).

**Figure 3:**
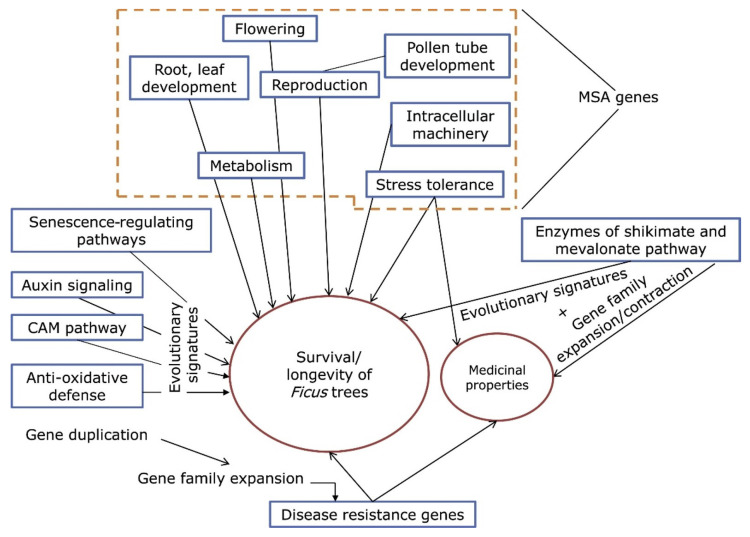
Evolutionarily significant biological processes responsible for *Ficus* longevity (modified Figure 3 in [9]).

Seventeen genes with multiple signs of adaptive evolution (MSA) found in *F. benghalensis* and nineteen such genes in *F. religiosa* (Table S1) were involved in functions essential for providing longevity in plants, such as root development, reproduction, metabolism, etc. (Figure 3) [9]. Interestingly, 15 out of 17 MSA genes in *F. benghalensis* were associated with stress tolerance, while in *F. religiosa*, 17 out of 19 MSA genes were.

**Figure 4:**
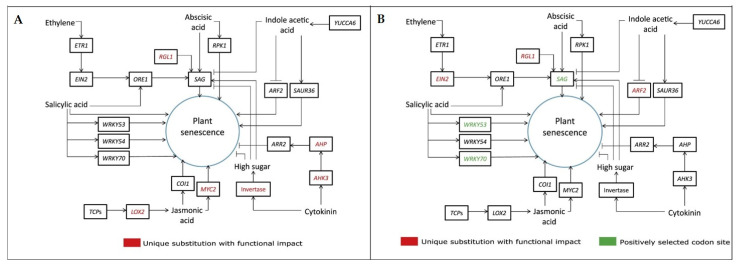
Adaptive evolution of genes involved in regulating senescence in *Ficus benghalensis* (**A**) and *F. religiosa* (**B**) (Figure 4C,D in [9]).

A comparative evolutionary analysis of these angiosperm species showed that the genes necessary for plant growth, development and stress resistance mechanisms are highly developed in these species, and may be responsible for the longevity of these two *Ficus* species [9]. The *AHK3*, *AHP*, *MYC2*, *RGL1*, *LOX2* and *Invertase* genes in *Ficus benghalensis* (Figure 4A) and the *EIN2*, *ARF2*, *RGL*, *SAG*, *WRKY53* and *WRKY70* genes in *F. religiosa* (Figure 4B) are likely involved in the regulation of aging and affect lifespan [9].

For example, *AHK2* and *AHK3* cytokinin receptor mutants with increased function have been shown to regulate plant organ size, flowering time and plant lifespan in *Arabidopsis thaliana* [10].

Analysis showed that highly extended gene families of both *Ficus* species are involved in plant disease resistance functions [9]. Notably, one of these families in both species was the *CC-NBS-LRR* class gene family, which is one of the best-characterized disease resistance families (R proteins) in plants [11].

Genes involved in signal transduction and glutamate metabolism required for resistance to oxidative stress [12] showed unique non-synonymous nucleotide substitutions with functional effects in the *GLR3.3* genes in *F. benghalensis* and 5-oxoprolinase in *F. religiosa* [9].

## 5. *Populus*

Although some perennial plants can live for centuries, the host–microbiome partnerships and interaction mechanisms underlying their longevity remain unclear. To fill this gap, scientists studied age-related changes in the composition of root metabolites, transcriptomes and the microbiome of *Populus tomentosa* trees aged from 1 to 35 years old [13].

Multi-omics network analysis demonstrated that the increased abundance of Actinobacteria with tree age was strongly associated with flavonoid biosynthesis. Using genetic approaches, it was demonstrated that the flavonoid biosynthesis regulator gene *Transparent Testa 8* is associated with the recruitment of flavonoid-associated Actinobacteria. Further inoculation experiments of Actinobacteria isolates indicated that their colonization could significantly improve the host’s phenotype. Site-directed mutagenesis revealed that the *hyBl* gene cluster, involved in biosynthesis of an aminocyclitol hygromycin B analog in Streptomyces isolate bj1, is associated with disease suppression [13].

**Figure 5:**
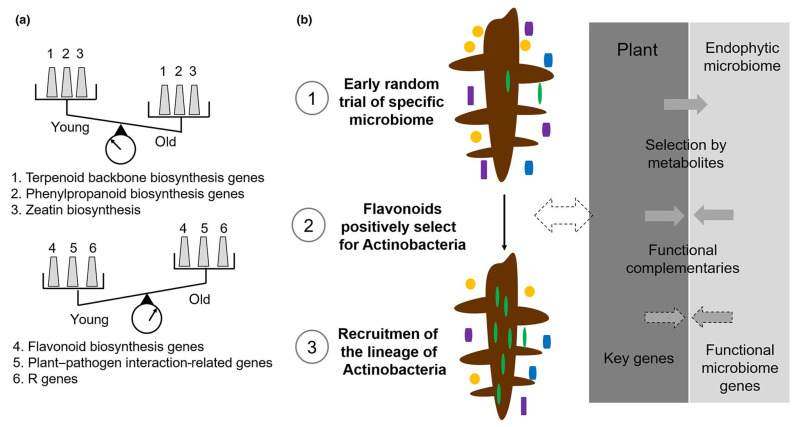
Schematic model showing the adaptation mechanisms underlying aging. (**a**) Dynamic patterns of some essential biological pathways underlying aging. (**b**) Proposed model for selection of Actinobacteria lineages during the development of poplar in the root-associated microbiome. (1) Early random trial of specific microbiome. (2) Altered metabolism positively selects for Actinobacteria. (3) Recruitment of the lineage of Actinobacteria. Dashed arrows indicate biological processes in the model and non-dashed arrows indicate regulating pathways between plant and microbiome (modified Figure 6 in [13]).

The growth of perennial plants is inherently limited by a number of pathogens and other stresses during their lifetime. Plants and the associated microbiota form a holobiont, wherein complex interactions and evolutionary selection contribute to the plant’s productivity and health, providing stability to the system. In one study, Actinobacteria were increasingly enriched in the root endosphere over the course of poplar development. The expression of a flavonoid metabolite gene significantly altered the microbial community on the roots of transgenic poplar trees. Accordingly, the activation of BGCs in the microbial partners could lead to disease suppression. This process may underlie ancient and mutually beneficial relationships between diverse hosts and microbes (Figure 5) [13].

## 6. *Welwitschia*

*Welwitschia* is famous for its longevity. Carbon-14 dating of some of the largest plants has shown that some individuals are over 1500 years old. The species has a highly distinctive morphology, consisting of just two leaves that grow continuously throughout the plant’s life. This can last several thousand years, resulting in the longest-lived leaves in the plant kingdom. Unlike other plants, the shoot apical meristem of *Welwitschia* dies in the young plant shortly after the appearance of true leaves and meristematic activity moves to the basal meristem [14].

**Figure 6:**
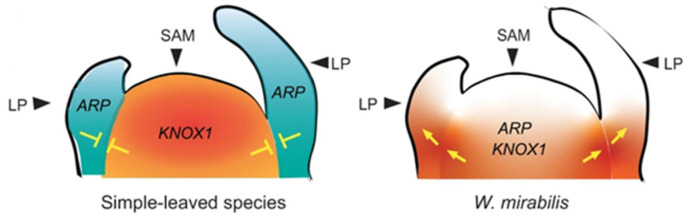
Diagrammatic representation of *KNOTTED-like homeobox Class 1* (*KNOX 1*) and *ASYMMETRIC LEAVES1/ROUGHSHEATH2/PHANTASTICA* (*ARP*) gene expression in a typical plant with determinate leaf growth (left) and in *Welwitschia* (right). With determinate leaf growth, there is an antagonistic regulation of KNOX 1 and ARP, which does not occur in *Welwitschia*, resulting in indeterminate leaf growth (SAM  =  shoot apical meristem, LP  =  leaf primordia) (Figure 4b in [14]).

Studies on *Welwitschia* have proposed that *KNOTTED-like homeobox Class 1* (*KNOX 1*) genes are expressed in the leaf base, causing a change in the mode of leaf growth from determinate to indeterminate [15]. The co-expression of *ASYMMETRIC LEAVES1/ROUGHSHEATH2/PHANTASTICA* (*ARP*) and *KNOX 1* genes in the shoot apical meristem and leaf primordia in Streptocarpus have also been linked to the extended leaf basal meristem activity in the development of unequal cotyledons [16]. Overlapping gene expression of *ARP3*, *ARP4* and *KNOX 1* was observed in the “basal meristem”, which is not observed in most simple-leaved species (Figure 6) [14].

**Figure 7:**
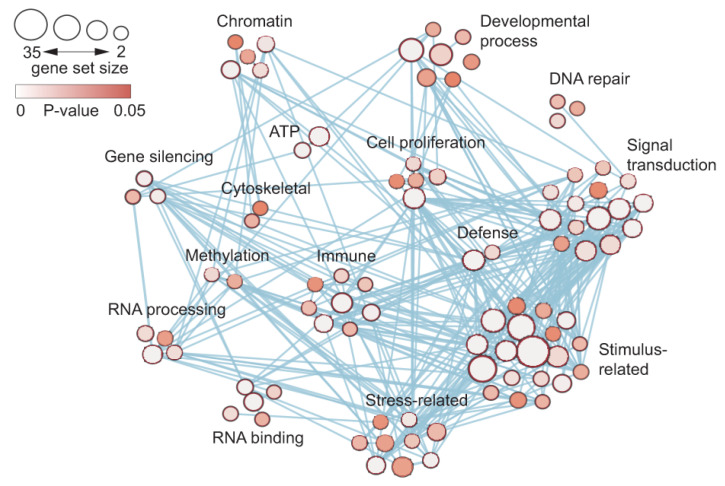
Weighted gene co-expression network analysis (WGCNA) revealed expression differences between basal meristems and young leaf material. The number of genes in each GO term enrichment category at each node are shown by the circle size. *p*-values are based on Fisher’s exact test, with two-sided and false discovery rate correction used for calculating *p* values. The probability that the node contains gene(s) that control differential gene expression between tissues is depicted by node color. The lines connecting each node indicate correlated gene expression between genes (Figure 4e in [14]).

To search for further signatures of indeterminate leaf growth, gene activity in the basal meristem compared with leaves was characterized using GO enrichment and weighted gene co-expression network analyses (WGCNA) (Figure 7). This analysis showed genes and pathways that are specifically co-expressed, and revealed the coordinated expression of genes involved in “stress-related” and “stimulus-related” response via the enhancement of “signal transduction”. These studies are consistent with the meristematic activity required for the continuous, indeterminate growth of *Welwitschia* leaves in environmentally stressful conditions [14].

It is assumed that the expansion of *R2R3-MYB* genes might be an adaptive response in *Welwitschia* for regulating cell division in the basal meristem to enable slow and continuous growth, tissue development and maturation over the long periods when environmental conditions are unfavorable. An expansion in the copy number of *HSP20* and *bHLH* gene family members, as well as upregulation of *NCED4,* was also found [14].

## 7. *Dracaena*

*Dracaena* species are monocotyledonous evergreen plants that are mainly distributed in tropical and subtropical regions of Asia and Africa. These are remarkably long-lived and slow-growing species and are renowned for their longevity. An analysis of several of the largest trees concluded that their age was likely to be several hundred years [17]. An analysis of the *D. cochinchinensis* genome identified evolutionary gene family expansions of the *small auxin upregulated RNA* (*SAUR*) genes and *cis-zeatin O-glucosyltransferase* (*cZOGT*) genes, which play important roles in growth retardation and the delay of senescence [18].

## 8. Epigenetic Mechanisms in *Pinus*

**Figure 8:**
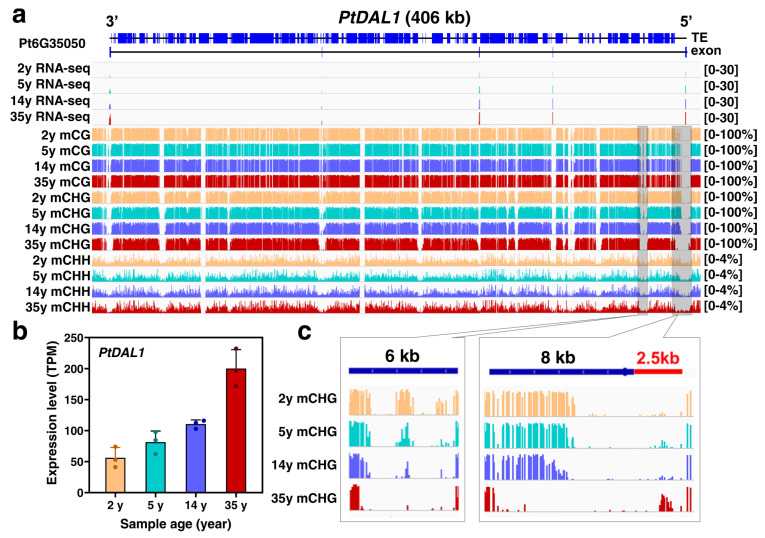
DNA methylation dynamics associated with the age timer *DAL1* age-related expression. (**a**) The DNA methylation patterns of the *PtDAL1* gene in four age stages. (**b**) The expression of *PtDAL1* at 2, 5, 14 and 35 years. Data were presented as means ± SD of three biological replicates. (**c**) Age-related CHG demethylation of *PtDAL1* (Figure 5 in [19]).

Li et al. [19] revealed that DNA methylation in *Pinus tabuliformis* may serve as an important epigenetic signature of developmental age in conifers. Two segments at the 5′-end of the first ultra-long intron in the *DAL1* gene [20] showed a gradual decline in CHG methylation as the age increased, which was highly correlated with its expression profile and can be considered as a conservative age biomarker in conifers (Figure 8).

**Figure 9:**
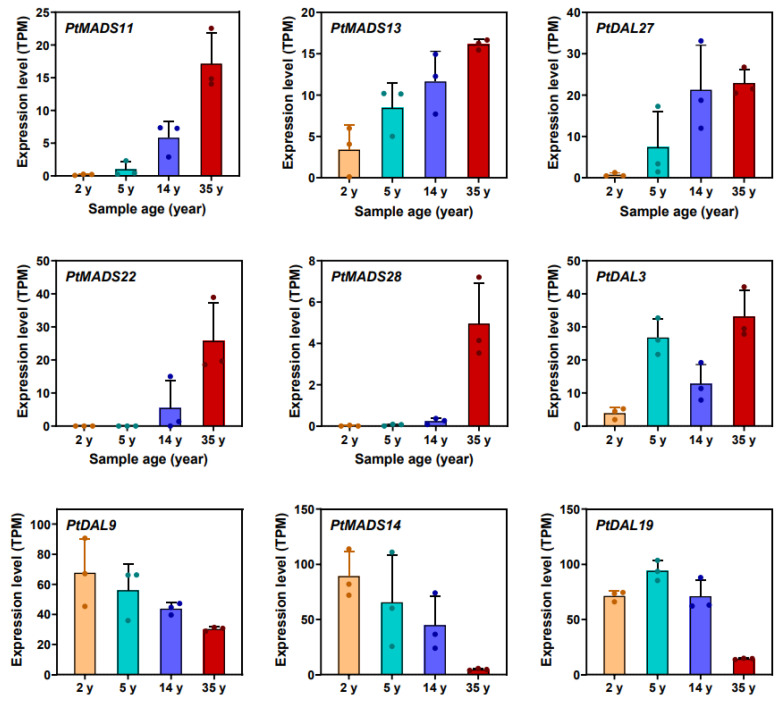
Expression changes of nine key *SOC1*-like transcription factors in the age-related gene module as the age increased. The data are presented as the means ± SD of three biological replicates, which values depicted as dots (Supplementary Figure 17 in [19]).

A similar high correlation was also observed in nine other age marker genes (*PtMADS11*, *PtMADS13*, *PtMADS14*, *PtMADS22*, *PtMADS28*, *PtDAL3*, *PtDAL19* and *PtDAL27*) (Figure 9) [19].

## 9. Traumatic Response to Wounds and Compartmentalization of Decay in Trees (CODIT)

Woody plants usually face injuries caused by different agents during their lives. The healing of injuries in the stem and branches, affecting the vascular cambium and xylem, can take several years. The secret of tree longevity is likely related to the ability of trees to isolate an invader and grow over it with appendage tissue (callus). This mechanism is activated whether it is a harmful insect, a pathogenic fungus or even a mechanical wound. Phenolic compounds (strong oxidants) are synthesized, and a chemical barrier is formed. The most important part is the outer wall, which is called No. 4, followed by No. 3 (the other side) and No. 1 and No. 2, which grow from above and below, respectively. This is called the compartmentalization of decay in trees (CODIT) [21,22].

**Figure 10:**
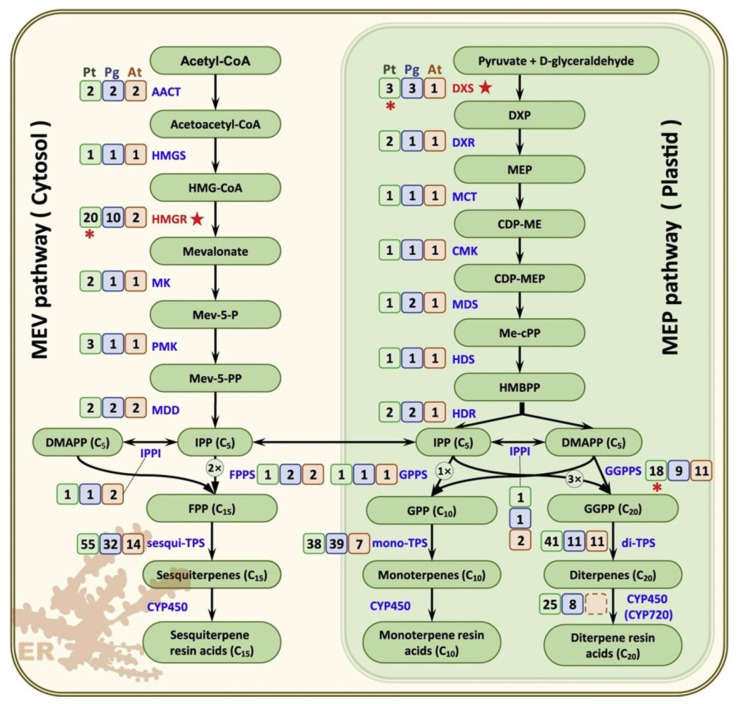
The resin terpene biosynthesis pathways in *Pinus tabuliformis*. The gene numbers of *P. tabuliformis* (green box), *Picea glauca* (blue box) and *Arabidopsis thaliana* (orange box) in the mevalonate (MEV) and methylerythritol phosphate (MEP) pathways. A five-point star represents the rate-limiting steps of isoprenoid biosynthesis. An asterisk denotes the genes that were duplicated in *P. tabuliformis*. AACT, acetyl-CoA acetyltransferase; HMGS, hydroxy methylglutaryl-CoA synthase; HMGR, hydroxy methylglutaryl-CoA reductase; MK, mevalonate kinase; PMK, phosphomevalonate kinase; MDD, diphosphomevalonate decarboxylase; DXS, 1-deoxy-d-xylulose-5-phosphate synthase; DXR, 1-deoxy-d-xylulose-5-phosphate reductoisomerase; MCT, 2-C-methyl-D-erythritol 4-phosphate cytidylyltransferase; CMK, 4-diphosphocytidyl-2-C-methyl-D-erythritol kinase; MDS, 2-C-methyl-D-erythritol 2, 4-cyclodiphosphate synthase; HDS, 2-C-methyl-D-erythritol 2, 4-cyclodiphosphate synthase; HDR, 2-C-methyl-D-erythritol 2, 4-cyclodiphosphate reductase; IPPI, isopentenyl-diphosphate delta-isomerase; GPPS, geranyl diphosphate synthase; FPPS, farnesyl pyrophosphate synthase; GGPPS, geranylgeranyl diphosphate synthase; TPS, terpenoids synthase; CYP450, cytochrome P450 (modified Figure 3A in [23]).

Resin exudation in conifers and wet wood in deciduous trees are also one of the first defense reactions. These mechanisms are likely also controlled by genes, but are not well-studied yet. However, 221 candidate genes encoding enzymes that catalyze the 22 enzymatic reaction steps of the resin terpene biosynthesis pathway were identified in *Pinus tabuliformis* (Figure 10, [23]).

Chano et al. [24] focused on the molecular basis of traumatic wood formation in *Pinus canariensis*, known for its extraordinary healing ability. Two main phases were identified during the first healing: the immediate response and development of traumatic wood, respectively. Wounding induced a complete rearrangement of the transcriptional program in the cambial zone close to the injuries. Initially, radial growth stopped, and a complete set of defensive genes, mostly related to biotic stress, were induced. Later on, cambial activity resumed in the lateral borders of the wound, even at a high rate. During this second stage, certain genes related to earlywood formation, including genes involved in cell wall formation and transcription factors, were significantly overexpressed, while certain latewood-related genes were suppressed. Such wood contained a high proportion of resin ducts, and also provided a good way to heal the wound in the shortest possible time [24].

## 10. *Larix kaempferi*

**Figure 11:**
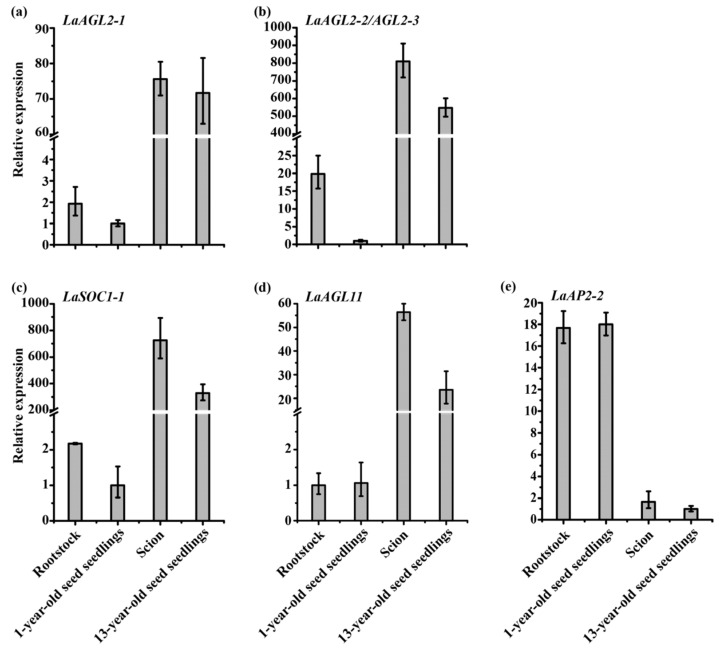
Expression patterns of the *LaAGL2-1* (**a**), *LaAGL2-2* (**b**), *LaAGL2-3* (**b**), *LaSOC1-1* (**c**), *LaAGL11* (**d**) and *LaAP2-2* (**e**) genes of *Larix kaempferi* in 3-month-old grafted seedlings (*n* = 6, sampled in 2019) and 1- and 13-year-old seed seedlings (*n* = 11, sampled in 2019) assayed using qRT-PCR with *LaFBP1* as the internal control (Figure 7 in [25]).

Based on a study of the expression patterns of 20 age-related transcription factors in *Larix kaempferi* trees subjected to grafting, cutting and pruning [25], it can be concluded that (1) cutting and pruning rejuvenated the plants and changed their gene expression, and the effects of cutting on gene expression were detectable within 14 years, although the cutting seedlings were still maturing during these years; (2) within three months after grafting, the rootstock was more sensitive to grafting than the scions and readily became mature with the effect of the scions, while the scions were not readily rejuvenated by the effect of the rootstock, because the expression of the *LaAGL2-1*, *LaAGL2-2*, *LaAGL2-3* and *LaSOC1-1* genes increased in the rootstock and did not decrease in the scion; (3) *LaAGL2-2* and *LaAGL2-3* were more sensitive to grafting, while *LaAP2-2* was impervious to it (Figure 11) [25].

## 11. Interspecific Traits of DNA Repair Genes Associated with Plant Longevity

Aoyagi Blue et al. [26] conducted a comparative genomic analysis of DNA repair genes for 61 plant species, including trees (angiosperms and gymnosperms), perennial and annual grasses and algae. Among 121 gene families, only 1 showed a significantly higher copy number ratio in tree species than in perennial and annual grass species, which was a poly(ADP-ribose)polymerase (PARP) involved in DNA repair [26].

The three species with the highest copy number ratio of *PARP* genes were Douglas fir (*Pseudotsuga menziesii*), Scots pine (*Pinus sylvestris*) and apple (*Malus domestica*). Douglas fir and Scots pine are known as long-lived conifers and can live for over 1000 years. Apple trees live between 60 and 100 years. Although the longevity of the apple tree is not as long as that of conifers, it is significantly longer than that of herb species [26].

It was checked whether there was a significant correlation between the copy number of *PARP* genes and life expectancy. Since the maximum lifespan of trees is unique and not always supported by scientific evidence, growth rate (height gain rate) was used as a proxy for lifespan.

**Figure 12:**
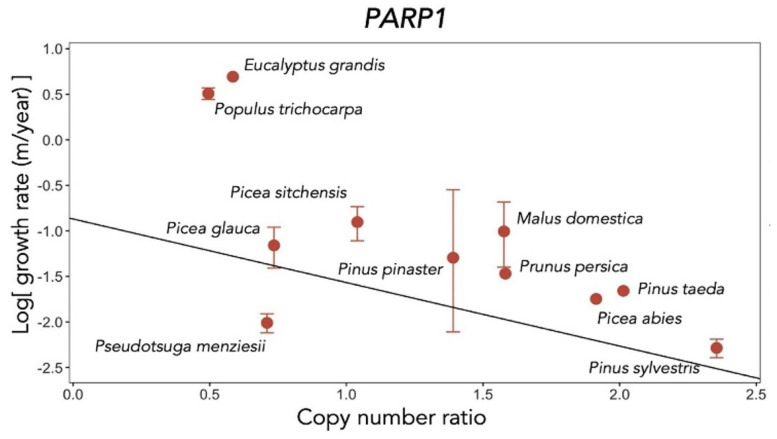
The relationships between growth rate and copy number ratio of *PARP1* in 11 tree species (Figure 5 in [26]).

There was a significant negative correlation between log growth rate (m/yr) and copy number ratio in the *PARP* gene family. Among the three members of the *PARP* family, a significantly negative correlation between log growth rate and copy number ratio was shown only for *PARP1* (Figure 12). This result strongly suggests an important role of *PAPR1* in the slow growth and longevity of tree species [26].

It is known that the pharmacological and genetic inhibition of PARP in *A. thaliana* leads to increased stress resistance and increased growth due to the prevention of cell death, but also leads to reduced protection due to a decrease in the accumulation of protective molecules, especially anthocyanin and ascorbate [27]. The antagonistic relationship between increased growth and decreased protection through PARP inhibition provides important insight into the long-standing ecological argument that slow-growing trees live longer than fast-growing trees [28]. Long-lived, late-succession species typically grow more slowly, invest more resources in defense connections and structural support and maintain lower levels of photosynthesis and respiration than short-lived, early succession species.

**Figure 13:**
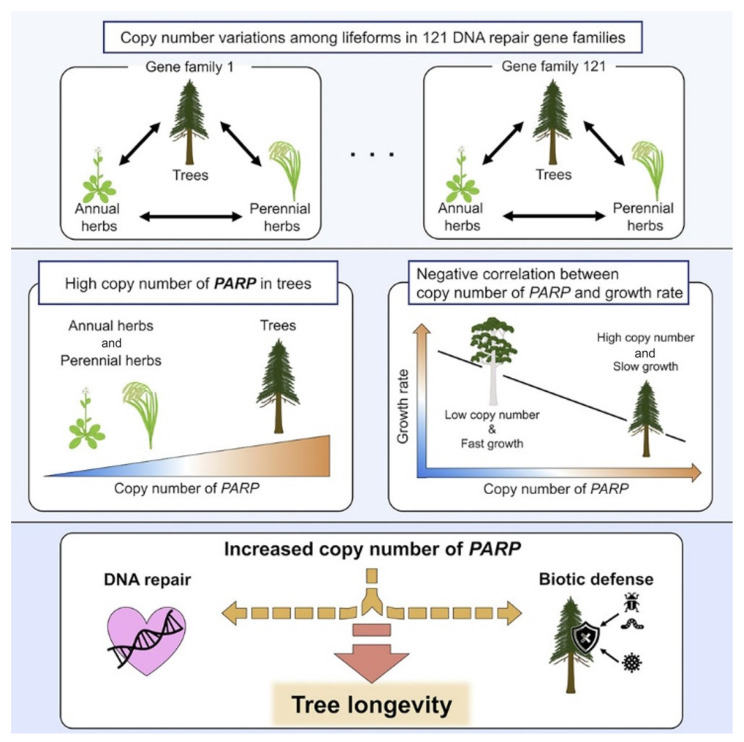
A general scheme for discovering genetic traits associated with the *PARP* gene family and plant longevity (graphical abstract in [26]).

Although the molecular mechanism underlying long-lived and short-lived tree species remains mostly unknown, one study puts forward a new, testable hypothesis that increasing the *PARP* copy number enhances the release of protective compounds, resulting in slow growth and a long lifespan. The general scheme of the study is shown in Figure 13 [26].

## 12. Interspecific Traits of Epigenetic Regulatory Genes Associated with Plant Longevity

A comparison of copy number variations in families of epigenetic regulatory genes among 85 plant species with different lifespans revealed an increase in the number of copies of *BRUSHY1/TONSOKU/MGOUN3* (*BRU1/TSK/MGO3*) and *SILENCING DEFECTIVE 3* (*SDE3*) genes in long-lived trees [29]. *BRU1/TSK/MGO3* is involved in chromatin modification and plays an important role in maintaining meristems and genome integrity. *SDE3* is involved in RNA silencing and plays an important role in antiviral defense through post-transcriptional gene silencing.

It is important to note that the specific defense mechanism of woody plants is the production of secondary metabolites that accumulate in the xylem and serve as a physical barrier against pathogens, which has been described as a very powerful defense mechanism [30].

It is known that DNA methylation patterns can reflect plant aging in the context of CpG, CHG and CHH methylation in *Pinus taeda* and represent an epigenetic clock capable of predicting the age of this species within 6% of its maximum lifespan. Although CHH methylation patterns showed little association with age, both CpG and CHG methylation patterns were strongly associated with aging, largely becoming hypomethylated with age. Among the age-associated loci were those that are in close proximity to the genes for malate dehydrogenase, NADH dehydrogenase and 18S and 26S ribosomal RNA [31].

In summary, the study [31] demonstrates an association between DNA methylation and chronological age in long-lived tree species and shows that changes in DNA methylation may be a universal indicator of aging.

## 13. The Impact of Developmental Phase Transition on Plant Longevity

Unlike animals, whose organs are typically formed during embryogenesis, vascular plants manage to extend their life by continuously producing new tissues and organs in apical and lateral directions via the proliferation of stem cells located within specialized tissues called meristems. Stem cells are the main source of plant longevity. Variation in plant longevity is highly dependent on the activity and fate identity of stem cells [32].

**Figure 14:**
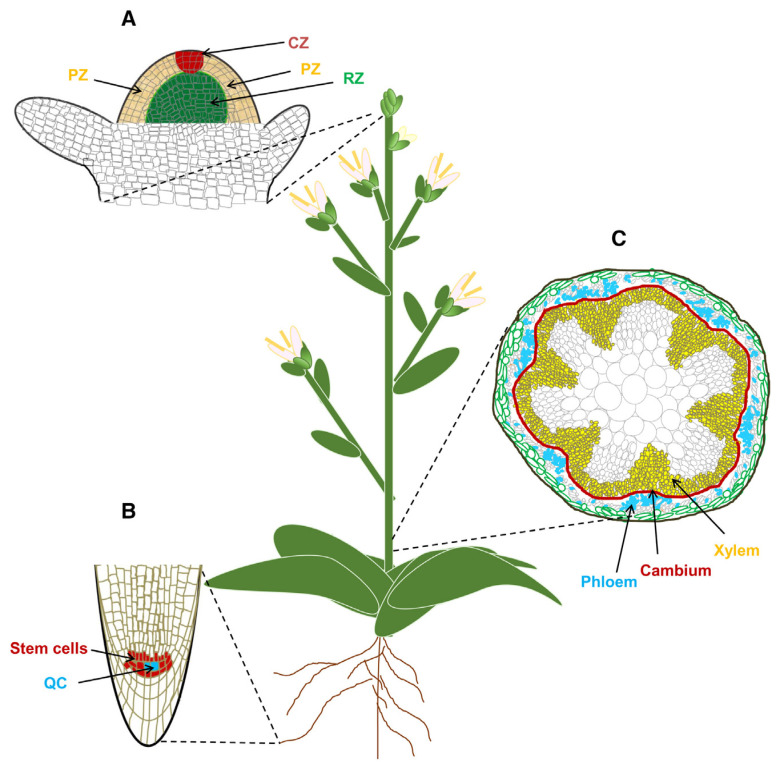
Stem cells in plants (Figure 1 in [32]). (**A**) Schematic of a longitudinal section of the shoot apical meristem (SAM) in Arabidopsis. The SAM consists of three developmental zones: (i) the central zone (CZ; red), with a population of slowly dividing stem cells; (ii) the surrounding peripheral zone (yellow), where cells divide rapidly to give rise to lateral organs, and (iii) the rib zone (green), where cells differentiate into central stem tissue. (**B**) Schematic of a longitudinal section of the root apical meristem (RAM) in Arabidopsis. The RAM consists of a small group of cells that form the quiescent center (QC; blue) and is surrounded by stem cells (red). Signals from the QC maintain the stem cell niche of the surrounding stem cells. (**C**) Schematic of a cross-section through the Arabidopsis inflorescence stem. The vascular cambium meristem (VCM) is shown in red. The vascular cambium generates the xylem (yellow) and phloem (blue) through inward and outward cell division, respectively.

In seed plants, almost all postembryonic production of tissues and organs results from the proliferation and differentiation of stem cells within meristematic tissues in the shoot apical meristem (SAM), root apical meristem (RAM) and vascular cambium meristem (VCM) (Figure 14) [32].

In seed plants, the SAM is responsible for continuously producing aboveground organs, including leaves, stems and flowers. The homeodomain transcription factor genes *WUSCHEL* (*WUS*) and *CLAVATA3* (*CLV3*), which encode a small functional peptide, are key factors that help the SAM to remain functional. Remarkably, the age-dependent cellular accumulation of reactive oxygen species in the *Arabidopsis* SAM shuts down stem cell activity, initiating programmed cell death [33].

The RAM also plays a crucial role in determining lifespan by ensuring the growth of the main root and, after branching, the lateral roots [32]. It is worth noting here that such particularities of roots in perennial plants as meristem indeterminacy, modular growth, stress resistance and patterns of senescence play the key role in perennial plant longevity [34]. The RAM encompasses the quiescent center (QC) and surrounding stem cells. The QC, as an organizer, is responsible for maintaining the surrounding stem cells. Transcription factors such as *ETHYLENE RESPONSE FACTOR 115*, *BRASSINOSTEROIDS AT VASCULAR AND ORGANIZING CENTER* and *SCARECROW* assist with activating the cell cycle machinery of QC cells in response to DNA damage-mediated death of root stem cells under environmental stress [32].

VCM is an important meristem whose activity gives rise to secondary growth, providing mechanical support and facilitating transport of water and nutrients throughout the plant body. The *TRACHEARY ELEMENT DIFFERENTIATION INHIBITORY FACTOR (TDIF)–PHLOEM INTERCALATED WITH XYLEM (PXY)–WUS*-related homeobox (*WOX*) signaling pathway is the best-understood pathway controlling cambium activity. The *TDIF*–*PXY* module plays an important role in the maintenance and proliferation of cambium cells by activating the expression of the cambium-specific *WOX4* and *WOX14* genes. The VCM remains viable throughout the lifespan of a woody plant [32]. The continuous activity of the VCM has been suggested as a key factor that may inhibit whole-plant senescence in woody plants [32,35].

## 14. The Effect of Axillary Meristems on Plant Longevity

The post-embryonic development and longevity of flowering plants are, for a large part, determined by the activity and maturation state of stem cell niches formed in the axils of leaves, the so-called axillary meristems (AMs). *AHL15* and other *AHL* clade-A genes play an important role, directly downstream of flowering genes (*SOC1*, *FUL*) and upstream of the flowering-promoting hormone gibberellic acid, in suppressing AM maturation and extending the plant’s lifespan [36].

The ability to maintain functional AMs after a successful round of offspring production is an important determinant of polycarpic growth behavior [37]. In *Arabidopsis*, TFL1 functions as a key regulator of inflorescence meristem indeterminacy and as a negative regulator of flowering time. Orthologs of *TFL1* also play important roles in determining whether AMs remain vegetative or commit to flowering in perennials, as demonstrated in *Malus domestica*, *P. trichocarpa*, *F. vesca* and *Lolium perenne* [32].

## 15. Polyploidy

It seems that polyploidy itself does not affect longevity. *Sequoia sempervirens* (D. Don) Endl. and *Sequoiadendron giganteum* (Lindl.) Buchh. are both record-holding long-lived species in the same family, Cupressaceae. However, while the first species is a hexaploid, the second one is a diploid. In fact, polyploidy is very rare among gymnosperms and occurs only in 5% of 685 gymnosperm taxa [38]. It is unknown in pines, which include Great Basin bristlecone pine (*Pinus longaeva* D. K. Bailey), which holds the absolute record for longevity. In general, polyploids are not more common among long-lived plants than among the short-lived ones.

## 16. Conclusions

This review provides readers with a brief summary of the currently available data on the genetic and epigenetic mechanisms of longevity in forest trees. The results of a comparative genomic analysis of long-lived tree species and the main genetic aspects of their longevity are summarized in Table 1. In *Quercus robur*, *Ginkgo biloba*, *Ficus benghalensis* and *F. religiosa*, a key trait associated with plant lifespan is the enhanced immune defense:

- in *Quercus robur*, this is manifested in the increase in gene families such as RLK, RLP and NLR;
- in *Ginkgo biloba*, the expression of R-genes and preformed genes is associated with maintaining resistance with age;
- in both *Ficus* species, gene families of the *CC-NBS-LRR* class, which is one of the most well-characterized disease resistance families in plants, were significantly expanded.

Cross-species genetic comparisons of plants revealed a high copy number ratio of the *PARP1* family genes, which are involved in DNA repair and also enhance the defense response. The highest ratio was found in *Pseudotsuga menziesii*, *Pinus sylvestris* and *Malus domestica*. There was also a significant negative correlation between log growth rate (m/yr) and *PARP1* copy number ratio.

A positive correlation was found between poplar age and the abundance of Actinobacteria, which may induce plant defense responses. The increase abundance of Actinobacteria with tree age was strongly associated with flavonoid biosynthesis. The regulatory *TRANSPARENT TESTA 8* (*TT8*) gene, which belongs to the *bHLH* gene family, may be a key regulator involved in the late steps of flavonoid biosynthesis with the potential to modulate the Actinobacteria abundance.

Studies on *Welwitschia* proposed that *KNOTTED-like homeobox Class 1* (*KNOX 1*) genes are expressed in the leaf base, causing a change in the mode of leaf growth from determinate to indeterminate. In addition, the *ASYMMETRIC LEAVES1/ROUGH SHEATH2/PHANTASTICA* (*ARP*), *R2R3-MYB*, *HSP20* and *NCED4* genes and *bHLH* gene family genes are associated with the activity of the basal meristem.

Analysis of the *Dracaena cochinchinensis* genome identified evolutionary gene family expansions of the *small auxin upregulated RNA* (*SAUR*) genes and *cis-zeatin O-glucosyltransferase* (*cZOGT*) genes, which play important roles in growth retardation and the delay of senescence.

An increase in the number of copies of the epigenetic regulators *BRU1/TSK/MGO3* (maintenance of meristems and genome integrity) and *SDE3* (antiviral protection) was also found in long-lived trees.

Two segments at the 5′-end of the first ultra-long intron in the *DAL1* gene in *Pinus tabuliformis*, a conservative age biomarker in conifers, showed a gradual decline in CHG methylation as the age increased and might indicate that epigenetic modifications such as methylation could also be associated with aging in plants.

The resistance of plants to various damage (chemical, biological, mechanical) can contribute to longevity. For example, a high number of resin ducts appear in traumatic wood in conifers, which is likely to be genetically controlled, but this is not well studied yet.

It was shown in larch that grafting, cutting and pruning change the expression of age-related genes and rejuvenate plants through currently unknown mechanisms.

In general, it seems that the expansion of some important gene families positively affects longevity by providing “genetic backup” for important functions, while polyploidy itself does not affect longevity.

## Figures and Tables

**Table 1:** Longevity mechanisms in long-lived trees.

Species (Family)	Normal Species Lifespan Range and Individual Tree Maximum Longevity, Years	Longevity Mechanisms
**Gymnosperms**		
*Ginkgo biloba* L. (Ginkgoaceae)	2000–2500, 4023 ^a^	high expression of disease resistance- and protective secondary metabolite-associated genes retained in old trees; continued expression of genes related to autophagy, senescence and age-related miRNAs; continued cambial divisions [3]
*Pseudotsuga menziesii* var. *menziesii* (Mirb.) Franco (Pinaceae)	400–600, 1350 ^b^	high copy number of genes in the DNA repair gene family *PARP* (poly(ADP-ribose) polymerase) [26]
*Pinus sylvestris* L. (Pinaceae)	150–300, 810 [39]	
*Pinus tabuliformis* (Pinaceae)	150–200, 481 [40], ~1000 ^c^	the *DAL1* gene, a conservative age biomarker in conifers, showing a gradual decline in CHG methylation as the age increased [20]; similar high correlation is also observed in nine other age marker genes (*PtMADS11*, *PtMADS13*, *PtMADS14*, *PtMADS22*, *PtMADS28*, *PtDAL3*, *PtDAL19*, and *PtDAL27*) [19]
*Larix kaempferi* (Pinaceae)	100–150, 200	20 age-related transcription factors (*LaCA*, *LaAGL2-1*, *LaAGL2-2*, *LaAGL2-3*, *LaSOC1-1*, *LaSOC1-2*, *LaSOC1-3*, *LaSOC1-4*, *LaAGL1*, *LaAGL11*, *LaAGL42*, *LaERF017*, *LaERF3*, *LaSCL29*, *LaAP2-1*, *LaAP2-2*, *LaHCA2*, *LaOZF2*, *LaTRL6* and *LaPHL1*) [25]
*Sequoiadendron giganteum* (Lindl.) J.Buchh. (Cupressaceae)	2000–3000, 3266 ^d^	high copy number of *BRUSHY1/TONSOKU/MGOUN3* (*BRU1*/*TSK*/*MGO3*) and *SILENCING DEFECTIVE 3* (*SDE3*) epigenetic regulatory genes in this and a few other long-lived trees [29]
*Welwitschia mirabilis* (Welwitschiaceae)	400–1500, 2000 ^e^	*KNOX1* genes are expressed in the leaf base, causing a change in the mode of leaf growth from determinate to indeterminate; the *ARP*, *R2R3-MYB*, *HSP20* and *NCED4* genes and *bHLH* gene family genes are associated with the activity of the basal meristem [14,15]
**Angiosperms**		
*Quercus robur* L. (Fagaceae)	300–500, 1637 ^f^	abundant *RLK*, *RLP* and *NLR* gene families [4]
*Ficus benghalensis* (Moraceae)	200–300, 550 [7,8]	17 genes with multiple signs of adaptive evolution (MSA), including 15 stress-related [9]
*Ficus religiosa* (Moraceae)	900–1500, 2217 ^b^	19 genes with MSA, including 17 stress-related [9]
*Dracaena cochinchinensis* *(Asparagaceae)*	several hundred years [17,18]	evolutionary gene family expansions of the *small auxin upregulated RNA* (*SAUR*) genes and *cis-zeatin O-glucosyltransferase* (*cZOGT*) genes [18]
*Populus tomentosa* (Salicaceae)	typically, 100–200 years, but sometimes over 500 years [41]	increased abundance of Actinobacteria was strongly associated with tree age and the flavonoid biosynthesis; the *TT8* gene may be a key regulator involved in the flavonoid pathway, with potential to modulate the Actinobacteria abundance [13]

^a^ https://www.monumentaltrees.com/en/trees/ginkgo/records (accessed on 14 February 2023). ^b^ http://www.rmtrr.org/oldlist.htm (accessed on 14 February 2023). ^c^ http://yllhj.beijing.gov.cn/English/Resources/202107/t20210720_2445104.html (accessed on 15 June 2023). ^d^ https://www.nps.gov/yose/learn/nature/sequoia-research.htm (accessed on 14 February 2023). ^e^ http://pza.sanbi.org/welwitschia-mirabilis (accessed on 14 February 2023). ^f^ https://en.wikipedia.org/wiki/Granit_oak (accessed on 14 February 2023).

## Data Availability

The data presented in this review are contained within the article and available in supplementary materials.

## Supplementary Materials

The following supporting information can be downloaded at: https://www.mdpi.com/article/10.3390/ijms241210403/s1.
