# Supplementary material for: Genetic and Epigenetic Mechanisms of Longevity in Forest Trees
Source: Int J Mol Sci. 2023 Jun 20;24(12):10403. doi: 10.3390/ijms241210403 (PMC10299211; doi:10.3390/ijms241210403)
Supplement: Supplementary file 1 [file ijms-24-10403-s001.zip › ijms-2425920-supplementary.pdf]

Table S1. Genes with multiple signs of adaptive evolution (MSA) in *Ficus benghalensis* and *F. religiosa* [8].

#	KEGG ID/*Arabidopsis* ortholog ID	Gene name
Ficus benghalensis		
1	AT1G31440	SH3P1 (SH3 domain-containing protein 1)
2	AT2G35100	ARAD1 (Arabinan Deficient 1)
3	AT1G08730	XIC (Myosin XI C)
4	AT5G12040	Nitrilase/cyanide hydratase and apolipoprotein N-acyltransferase family protein
5	AT5G41040	Aliphatic suberin feruloyl-transferase
6	AT1G80410	NAA15 (N-Alpha-Acetyltransferase 15)
7	AT1G16880	ACR11 (ACT domain repeats 11)
8	AT4G10380	NIP5;1 (NOD26-like Intrinsic Protein 5;1)
9	AT1G22540	NPF5.10 - Major facilitator superfamily protein
10	AT5G15810	TRM1B (tRNA methyltransferase 1B)
11	AT2G38560	RDO2 (Reduced dormancy 2)
12	K13348	Protein MPV17
13	AT5G24690	Plant/protein, putative (DUF3411)
14	AT5G15900	TBL19 (Trichome birefringence-like 19)
15	AT3G21180	ACA9 - Autoinhibited Ca(2+)-ATPase 9
16	AT5G64816	Thionin-like gene
17	K00134	GAPDH (Glyceraldehyde 3-phosphate dehydrogenase)
Ficus religiosa		
1	AT1G79000	HAC1 (Histone Acetyltransferase 1)
2	AT4G04910	NSF (N-Ethylmaleimide sensitive factor)
3	AT3G02130	RPK2 (Receptor-like protein kinase 2)
4	AT3G63190	HFP108
5	AT5G65310	HB5 (Homeobox protein 5)
6	AT1G08320	TGA9 (TGACG (TGA) motif-binding protein 9)
7	AT3G19860	BHLH121 (Basic helix-loop-helix 121)
8	K00134	GAPDH (Glyceraldehyde 3-phosphate dehydrogenase)
9	AT1G63430	Leucine-rich repeat protein kinase family protein
10	AT2G43710	SSI2 (Suppressor of SA Insensitive 2)
11	AT3G62240	SRH1 (RING/U-box superfamily protein)
12	AT1G10200	WLIM1 (LIM domain-containing protein)
13	AT4G02280	SUS3 (Sucrose synthase 3)
14	AT2G28500	LBD11 (LOB domain-containing protein 11)
15	AT2G39210	PIC30 (Picloram Resistant 30)
16	K02218	Casein kinase 1
17	AT5G41970	Metal-dependent protein hydrolase
18	K20891	β-glucuronosyltransferase
19	AT1G05010	ACO4 (1-Aminocyclopropane-1-Carboxylic Acid Oxidase)
